# Abundant, distinct, and seasonally dynamic bee community in the canopy‐aerosphere interface above a temperate forest

**DOI:** 10.1002/ece3.9739

**Published:** 2023-02-15

**Authors:** Michael J. Cunningham‐Minnick, Joan Milam, Brian Kane, H. Patrick Roberts, David I. King

**Affiliations:** ^1^ Department of Environmental Conservation University of Massachusetts Amherst Massachusetts USA; ^2^ U.S. Forest Service Northern Research Station Amherst Massachusetts USA

**Keywords:** blue vane trap, canopy‐aerosphere interface, native bee, temperate forest, tree resources, vertical strata

## Abstract

Our understanding of how bees (Apoidea) use temperate forests is largely limited to sampling the understory and forest floor. Studies over the last decade have demonstrated that bee communities are vertically stratified within forests, yet the ecology of bee assemblages immediately above the canopy, the canopy‐aerosphere interface, remains unexplored. We sampled and compared bee communities above the canopy of a temperate forest to the understory (1 m), midstory (10 m), and canopy (20 m) on the campus of the University of Massachusetts, in Amherst, Massachusetts, United States from April to August, 2021. Overall, we found that assemblages above the canopy had more bees than in the understory, were distinct in composition from all other strata, and included the greatest proportion of unique species. Bee abundance and species richness were highest in the understory throughout the spring (April and May) and decreased as the season progressed, while bee abundance and species richness at higher strata increased into the summer months. We also found that bees with preferences to nest in moist and rotting wood were largely restricted to canopy and midstory strata. We conclude that bee assemblages occupying the space above the forest canopy are abundant and diverse, and their unique composition suggests that this canopy‐aerosphere interface plays an additional role in the bee community of temperate forests. Alternatively, our findings question how forest bee communities should be defined while highlighting the need for research on fundamental processes governing species stratification in and above the canopy.

## Graphical abstract

Vertical stratification of bee communities in temperate forests extends into the aerosphere, but the assemblage above the canopy is distinct from those within the forest. Most bees occupy the understory when floral resources of the forest are abundant in spring, but the bee community shifts into and above the canopy as the summer progresses and floral resources of the forest senesce. Examination of species life‐history traits demonstrated that wood‐nesting species remained within forest strata, while soil‐nesting species that were either males or females associated with open habitat, comprised the canopy‐aerosphere interface.

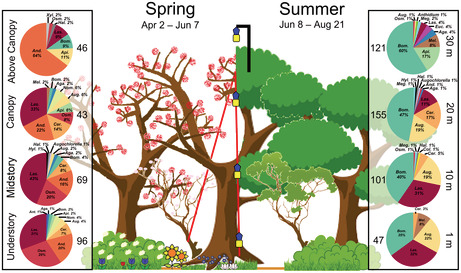

## 1 INTRODUCTION

Studies examining bee communities within temperate forests have largely restricted sampling to the understory (Milam et al., 2022) with the presumption that most bees remain in this lower stratum. However, recent evidence indicates that bees are vertically distributed within temperate forests (e.g., Allen & Davies, 2022; Ulyshen et al., 2010; Urban‐Mead et al., 2021), suggesting a potentially large knowledge gap in the ecological role of these important forest pollinators. Despite this revelation, research regarding the vertical distribution of bees and other pollinators within forests is further limited by the difficulty of sampling the high canopy (Cannon et al., 2021; Cunningham‐Minnick et al., 2022). Current sampling methods reach into the canopy (e.g., Maguire et al., 2014; Ulyshen et al., 2010), but the canopy‐aerosphere interface—a potentially ecologically important area for bees due to copious floral resources available—remains unexplored in temperate forests (Nakamura et al., 2017; Urban‐Mead et al., 2021). Thus, our understanding of pollinator ecology within forests will remain incomplete until the distribution of forest bee communities along the entire vertical gradient of vegetation structure is documented. Moreover, if the current understanding of bee abundance and diversity patterns in forests is inaccurate, forest management recommendations for bee conservation may be biased or potentially misguided (Milam et al., 2022; Urban‐Mead et al., 2021), further highlighting the importance of understanding the distribution of bee communities along the full vertical gradient of temperate forests, including the canopy‐aerosphere interface.

Bees are expected to be spatially and temporally distributed throughout temperate forests in response to local resource availability. Studies have demonstrated that forest bee communities are diverse and vertically stratified on sun‐exposed edges (e.g., Allen & Davies, 2022; Cunningham‐Minnick & Crist, 2020) and within the forest interior (e.g., Allen & Davies, 2022; Campbell et al., 2018; Milam et al., 2022; Ulyshen et al., 2010; Urban‐Mead et al., 2021) when floral resources of the forest are available, as well as when they are not. Inferences and observations further suggest that bees will both forage on floral resources and nest at different vertical strata within forests (Allen & Davies, 2022; Cunningham‐Minnick & Crist, 2020; MacIvor et al., 2014; Russo & Danforth, 2017; Smith et al., 2019; Sobek et al., 2009; Urban‐Mead et al., 2021; Wood et al., 2018). For instance, Smith et al. (2019) and Wood et al. (2018) found support through pollen analyses that forest bee communities rely upon floral resources of dominant tree species. Yet floral resources of herbaceous and woody species within temperate forests are typically limited to spring and early summer phenology, which has been correlated to fewer late‐season bees in the forest understory (Cunningham‐Minnick & Crist, 2020). Alternatively, studies have found more bees in the forest herbaceous layer during spring and more bees in the canopy during the summer (Cunningham‐Minnick & Crist, 2020; Ulyshen et al., 2010), suggesting that the distribution of forest bees may also shift out of the understory and into the higher vertical strata of the forest as the year progresses. However, no studies have compared the bee fauna in the aerosphere above the forest canopy to strata within temperate forests. Thus, we undertook this study to determine the extent to which bees occupy the open air above the forest canopy, how the bee assemblages of this canopy‐aerosphere interface compare in abundance, species richness, and composition with assemblages at other strata, and how these patterns change with seasonal phenology.

## 2 MATERIALS AND METHODS

**FIGURE A1:**
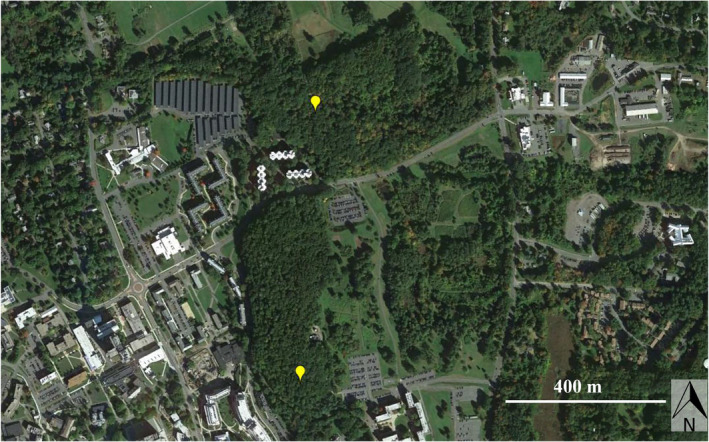
Aerial photo of the two forest fragments within the study area. Yellow icons mark locations of sites. The bee community was sampled at one *Acer rubrum* and one *Quercus rubra* tree within each site.

We selected two trees ⁓50 m apart in each of two forest patches on the campus of the University of Massachusetts‐Amherst in Amherst, Mass., USA (Figure A1); each pair of trees consisted of a northern red oak (*Quercus rubra* L.) and a red maple (*Acer rubrum* L.). Both sites were in USDA Hardiness Zone 5a and were characterized by an herbaceous stratum of ferns (e.g., *Dennstaedtia punctilobula* (Michx.) T. Moore, *Polystichum acrostichoides* (Michx.) Schott), wild sarsaparilla (*Aralia nudicaulis* L.), white wood aster (*Eurybia divaricata* (L.) G. L. Nesom), star flower (*Lysimachia borealis* (Raf.) U. Manns & Anderb), Canada mayflower (*Maianthemum canadense* Desf.), partridge berry (*Mitchella repens* L.), and Solomon's seal (*Polygonatum* spp.). The understory of these sites consisted of brambles (*Rubus* spp.), poison ivy (*Toxicodendron radicans* (L.) Kuntze), maple‐leaf viburnum (*Viburnum acerifolium* L.), witch hazel (*Hamamelis virginiana* L.), glossy buckthorn (*Rhamnus cathartica* L.), and seedlings of the dominant canopy trees (e.g., *A. rubrum*, *A. saccharum* Marshall, *Betula lenta* L., *B. papyrifera* Marshall, *Q. rubra* and *Q. alba* L.). We chose *A. rubrum* and *Q. rubra* because they are dominant species in forests of the area and represent different flowering systems, blooming times, and floral resource availability that span the blooming duration of most nearby trees. For instance, *A. rubrum* produces showy flowers rich in nectar and pollen early in the spring, while *Q. rubra* flowers are not showy but instead heavy with pollen and appear after the senescence of *A. rubrum* flowers (Batra, 1985; Cunningham‐Minnick et al., 2022). We chose these forest patches due to their accessibility and general representation of dominant species in forests of the area.

**FIGURE 1:**
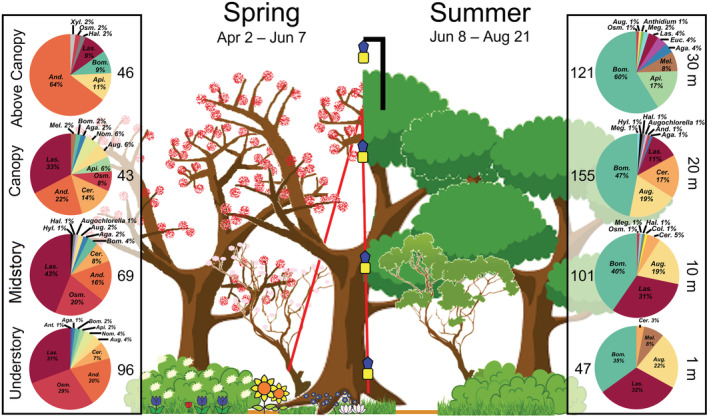
Sampling trap design and composition (first three letters of genus) for the bee community at 1, 10, 20, and 30 meters above the ground with blue vane traps to represent the understory, midstory, canopy, and above the canopy of the forest, respectively, in the spring when floral resources were available and in the summer at full leaf‐out when floral resources were depleted. *Augochlorella* and *Anthidium* genera are unabbreviated to differentiate from *Augochlora* (*Aug*) and *Anthidiellum* (*Ant*), respectively. Traps in the understory, midstory, and canopy were attached to a rope hung over a high branch in the canopy and anchored to a nearby stem for easy collection. The trap above the canopy was employed using a telescoping hanger designed as described in Cunningham‐Minnick et al. (2022), which had a rope threaded through the hanger that was anchored to the stem in the understory to allow the trap to be lowered along with another cord at the trap to aid in lowering (not depicted). Numbers next to pie charts represent total abundance across sites.

The bee community was sampled using blue vane traps in the understory, midstory, canopy, and above canopy strata of the forests at each focal tree. Three traps were individually attached to a rope hung over a high branch in the canopy as in Cunningham‐Minnick and Crist (2020). Traps were placed 1, 10, 20, and ⁓30 m above the ground (Table A1) to represent the following strata: understory, midstory, canopy and above canopy (Figure 1). The trap above the canopy was set 1 m above the tallest leaf‐bearing branch of each tree using a telescoping hanger attached to a vertical limb in the crown of the canopy as described in Cunningham‐Minnick et al. (2022). Traps were deployed on April 2, 2022, and checked every 1–3 weeks until August 21, 2022, for a total of 12 checks. Bees were sorted, pinned, and identified to species by JM using published keys (e.g., Gibbs, 2011; Gibbs et al., 2013; LaBerge, 1987, 1989; Mitchell, 1960, 1962) and the online source Discoverlife.org (Ascher & Pickering, 2020); vouchered specimens are located at the Natural History Collections at the University of Massachusetts in Amherst, MA. To distinguish differences in microclimate from other conditions among strata, Onset HOBO® Pendant data loggers (Part AU‐002‐64) were placed directly above each trap to record the light intensity and temperature every 10 min from June 7–21, 2022, to provide data on daily microclimate conditions and hourly from June 22–August 21, 2022, to represent seasonal change.

### 2.1 Data analysis

To compare bee abundance and species richness across vertical strata throughout the sampling season, we built generalized linear mixed effects models with negative binomial errors and created 95% confidence intervals of pairwise comparisons for each response across strata. All analyses were performed in the R statistical software (R Core Team, 2021). Models were made using the glmmTMB function in the glmmTMB package (Brooks et al., 2017) with fixed effects of stratum (understory, midstory, canopy, above canopy), sample (1–12) as a continuous variable, and their interaction. We allowed the model intercept to vary by each unique tree from which traps were hung to account for tree‐ and location‐specific differences. We also included an offset term of the log of the trap deployment duration (days) to account for differences in sampling effort. The significance of interaction terms was evaluated by likelihood ratio tests; simulated model residuals through the DHARMa package were used to evaluate the overall model fit (Hartig, 2020). Post‐hoc comparisons were made using the confint and glht functions in the multcomp package (Hothorn et al., 2008). Differences in bee species composition among strata were visualized with nonmetric multidimensional scaling ordinations performed on a species occurrence matrix of Sorensen distances using the metaMDS function in the vegan package (Oksanen et al., 2019); statistics and *p*‐values were derived using the pairwiseAdonis function with a Bonferroni adjustment for multiple comparisons (Arbizu, 2017). To align our sampling design with the ecological processes of the study area, we considered bees encountered after June 7 as associated with summer conditions. For instance, floral resources were abundant and the canopy was open throughout the vertical gradient of the forest prior to this date but not after (Figure 1).

## 3 RESULTS

**FIGURE 2:**
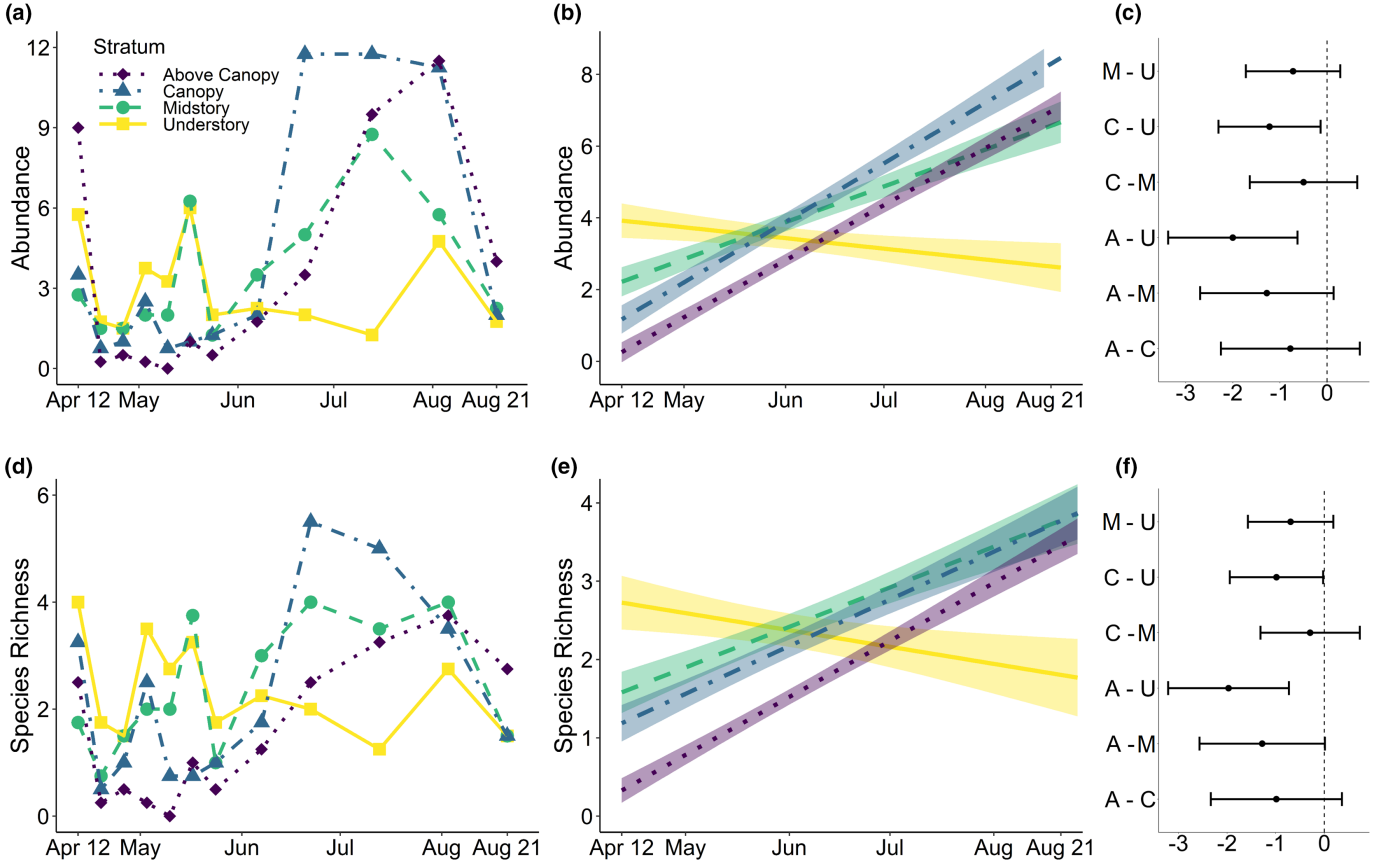
Relationships between bee abundance (a–c) or species richness (d–f) and time of year among strata, including mean values of the data (a and d), fitted mixed effects models with 95% CI (b and e), and pairwise contrasts (logged response) between strata (c and f): Above canopy (A), Canopy (C), Midstory (M), and Understory (U).

**FIGURE A2:**
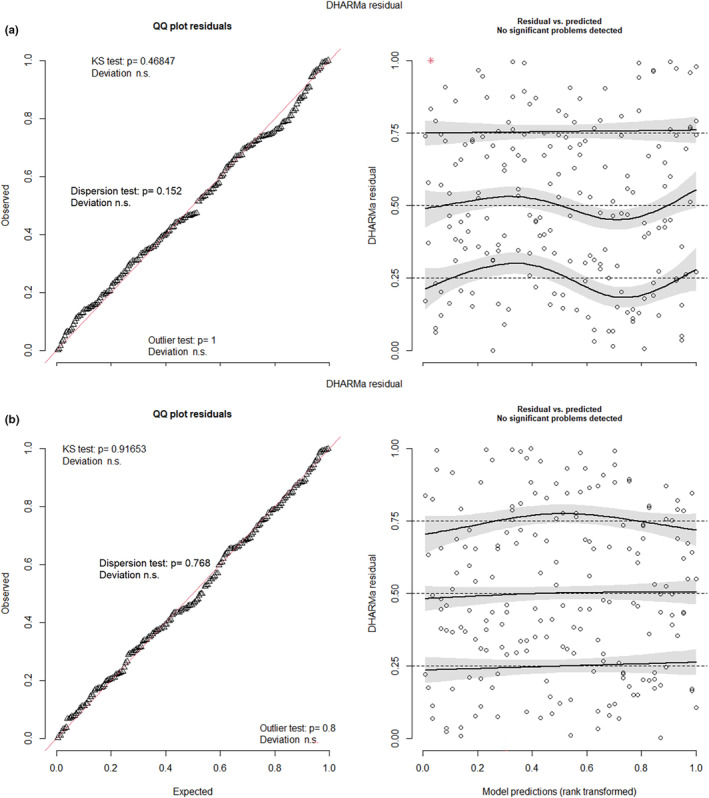
Examination of residuals for proper model fit for bee abundance (a) and species richness (b). Output from the DHARMa package in R.

We collected 144 bees representing 37 species in the understory, 170 bees from 31 species in the midstory, 198 bees consisting of 36 species in the canopy, and 167 bees from 28 species in the aerosphere above the canopy, for a total of 679 bees representing 75 species across strata (Table A2; full details in Data.xlsx of supporting information). Twelve specimens could not be identified to species due to body damage and were not included in species richness or composition analyses. After accounting for differences among individual trees, generalized linear mixed models found that there were significantly more bees and bee species in the understory than within, or above, the canopy (Figure 2c,f). Interaction terms (abundance: χ^2^(3) = 19.0, *p* < .0005; richness: χ^2^(3) = 16.4, *p* < .001) demonstrated that bee abundance (χ^2^(7) = 24.1; *p* < .005) and species richness (χ^2^(7) = 30.8; *p* < .0001) changed among strata throughout the study period (Figure 2b,e; Figure A2). Specifically, bee abundance and species richness were highest within the understory during the spring months (April and May) and decreased as the season progressed, while more bees and more species were encountered in and above canopy layers during the summer months (Figures 1 and 2a,d).

**FIGURE A3:**
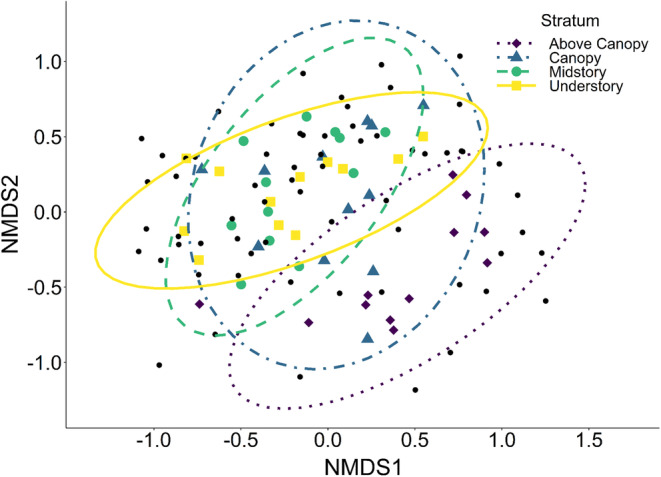
Nonmetric multidimensional scaling of bee community composition at each stratum, marked by colored dots and 95% confidence ellipses. Black dots represent species scores.

**FIGURE A4:**
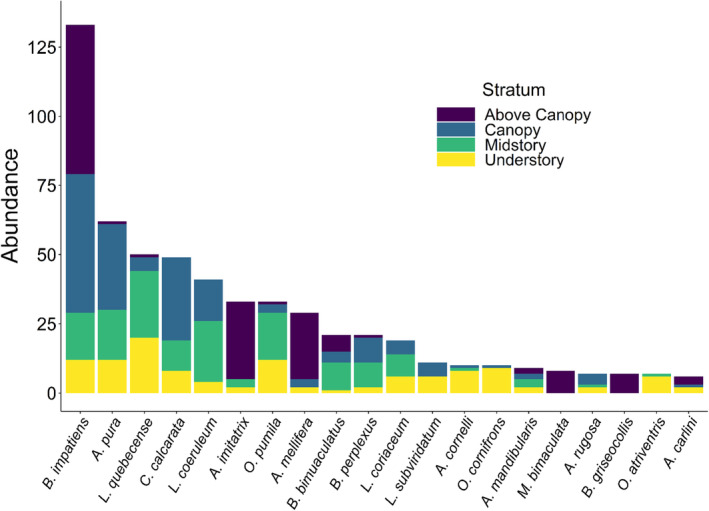
Stacked bar plot of bee abundances among forest strata for all bee species with >5 total individuals collected.

**FIGURE A5:**
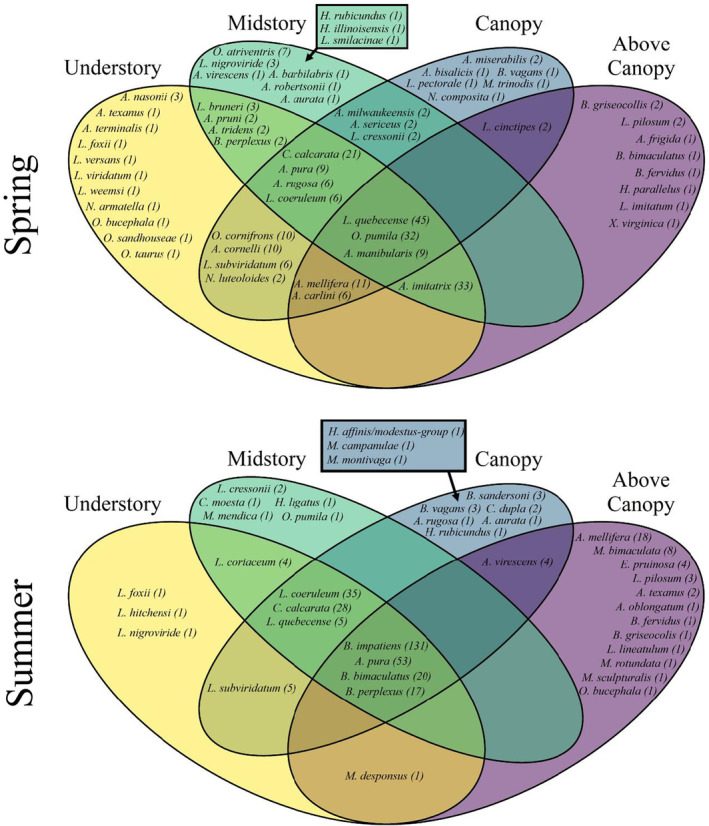
Spring (April 2–June 7) and summer (June 8–August 21) bee community composition at 1 m (Understory), 10 m (Midstory), 20 m (Canopy), and 30 m (Above canopy) above the forest floor. Number in parentheses represents total abundance. Species in overlapping ellipses were at multiple strata.

Species composition of the bee community above the canopy was significantly different from the understory, midstory, and canopy layers (Figure 1), but there were no statistical differences among the lower strata (Table A3; Figure A3). For instance, the most abundant (>10% relative abundance) bee genera in the spring months above the canopy were *Andrena* and *Apis*, while *Lasioglossum*, *Andrena*, *Osmia*, and *Ceratina* were common of strata within the forest. Similarly, *Bombus* and *Apis* were most abundant above the canopy during the summer months, while *Bombus*, *Lasioglossum*, *Augochlora*, and *Ceratina* bees were commonly encountered in lower strata. It was also found that abundant species were collected across strata, whereas 13 species occurred only above the canopy (Table 1; Figures A4 and A5).

**TABLE 1:** Total bee abundance and species richness, as well as number of unique species, females, soil‐nesting species, soil‐nesting individuals, wood‐nesting species that prefer moist and decayed (“soft”) wood, and soft wood‐nesting individuals at each stratum

	Understory	Midstory	Canopy	Above canopy
Total Abundance	144	170	198	167
Total Species Richness	37	31	36	28
Unique Species	10	7	11	13
Females	78%	82%	88%	78%
Soil Species	35%	28%	32%	28%
Soil Nester Abundance	59%	53%	55%	81%
“Soft” Wood Species	8%	10%	11%	4%
“Soft” Wood Nester Abundance	14%	25%	26%	1%

## 4 DISCUSSION

Our study is one of the first to demonstrate that bees occupy the aerosphere immediately above the canopy in temperate forests; furthermore, the community above the canopy was compositionally distinct with similar abundances compared with lower strata (understory, midstory, canopy). These findings expand our understanding of forest bee communities and build on earlier research that revealed differences between understory and canopy bees (Allen & Davies, 2022; Cunningham‐Minnick & Crist, 2020; Milam et al., 2022; Ulyshen et al., 2010; Urban‐Mead et al., 2021). However, when attempting to characterize the forest bee community, the importance of sampling the canopy‐aerosphere interface hinges upon the question of whether these bees should be considered as part of the forest community, or if they are transients moving among resources. The fact that the above canopy assemblage was generally characterized by many species that were not observed at lower strata and were also associated with nonforested habitats (e.g., *Agapostemon texanus* Cresson, *Halictus parallelus* Say, *Eucera pruinosa* (Say); Harrison et al., 2018) suggests that while some bees may forage on floral resources available at tree crowns in the spring, many others may be moving over the forest to access other habitat patches or resources throughout the season, as reported in other insect taxa (Wainwright et al., 2017). We report our findings as broad collective patterns of multiple species and caution against making species‐specific inferences since this was a pilot study with limited data. Nevertheless, the presence of an abundant and species‐rich bee assemblage at the canopy‐aerosphere interface, which had not previously been prioritized, suggests that more studies are needed to address the extent to which these bees should be considered a subset of the forest bee community.

**FIGURE A6:**
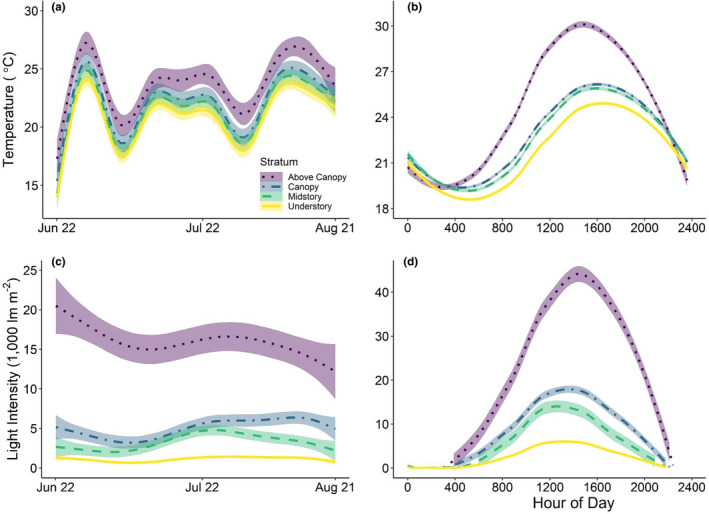
Generalized additive regressions of mean temperatures (a, b) and light intensities (c, d) of traps at each stratum with 95% CI. Readings were recorded in 60‐min intervals from June 22 to August 21 (a, c). Records were taken at 10‐min intervals (b, d) from June 7 to 21 to represent a typical 24‐h (*x*‐axis) day.

Our study also demonstrated how the vertical stratification of forest bee communities changed throughout the flight season. Our observations confirm the findings of previous studies that have documented a positive relationship between day of year and bee abundance in the canopy (i.e., Cunningham‐Minnick & Crist, 2020; Ulyshen et al., 2010; Urban‐Mead et al., 2021). However, while this pattern may reflect a response to the reduction in floral resource availability near the ground in temperate forests (Proesman et al., 2019; Ulyshen et al., 2010), it remains unclear why bees moved to the canopy. It is possible that the canopy provides alternative foraging resources (Campbell et al., 2018; Ulyshen et al., 2010), nesting opportunities (Cunningham‐Minnick & Crist, 2020), or favorable conditions associated with tree leaf phenology (Urban‐Mead et al., 2021). Our results add a layer of complexity to the issue by demonstrating that this temporal pattern extends vertically beyond the forest canopy and involves a compositionally distinct subset of the bee community that may be responding to a mix of environmental cues. For example, the highest bee abundance across sampling points in the spring was at the canopy‐aerosphere interface during the peak bloom of *A. rubrum*, suggesting that this stratum may provide access to the floral resources of the forest canopy. However, it seems unlikely that summer bees above the canopy were foraging or nesting since there were negligible forest floral resources and most bees were soil‐nesting species. Vegetation height has been negatively associated with bee abundance and diversity (Roberts et al., 2017); therefore, bees may instead use the canopy‐aerosphere interface for movement or dispersal since this space lacks the obstacles created by the vegetation structure of forest interiors. Alternatively, bees may be physiologically driven to take advantage of the greater light intensities and warmer temperatures above the canopy compared with other strata to forage earlier or later in the day (Figure A6; Kebler & Somanathan, 2019; Roubik, 1993). It is also possible that some species were seeking mates above the canopy. For instance, groups of male *Apis mellifera* L. mate with females 10–40 m above the ground (Ruttner, 1966); similarly, male groups of some *Bombus* species will fly to higher elevations to mate with emerging females, a behavior known as “hill‐topping” (Goulson et al., 2011). Though *A. mellifera* and *Bombus* spp. comprised 56% of the overall abundance of bees above the canopy, mating behaviors are unlikely to explain our findings because only three individuals of these species were males and three were reproductive females (all *Bombus*). These data also suggest there was no risk of oversampling the important genus of pollinator *Bombus* through the continuous deployment of blue vane traps in our study design. However, we did not quantify the density of reproductive females in this, or any other, genus in the study area and recognize that there were likely many more bee species within and above the forest canopy that would be revealed with additional trap types (Prendergast et al., 2020). We also terminated sampling at the end of August due to large declines in bee abundance and species richness observed throughout forest strata. Therefore, it is possible that additional patterns associated with common forest bees, which occur later in the year, such as mating of *Bombus* spp., were not observed. We also found that males of two solitary soil‐nesting species, *Andrena imitatrix* Cresson and *A. mandibularis* Robertson, comprised 57% of bee abundance above the canopy in the spring. We are not aware of any studies addressing mating behaviors similar to hill‐topping in these species or the genus *Andrena*. However, Urban‐Mead et al. (2022) found that male *A. imitatrix* consumes pollen of forest species (Urban‐Mead et al., 2022), including *A. rubrum* (personal communication), a tree species over which we encountered 93% of all *A. imitatrix* males in the dataset. Thus, there are many potential mechanisms that need to be tested to explain the occurrence of each species encountered above the forest canopy.

There were notable differences in bee assemblages among the other strata that may be best explained through life‐history traits. For instance, bees in our study that nest in moist, decayed wood (e.g., *Augochlora pura* (Say), *Lasioglossum coeruleum* (Robertson), *L. cressoni* (Robertson), *L. subviridatum* (Cockerell)) or pithy twigs (e.g., *Hylaeus* spp., *Ceratina* spp.) were nearly absent above the canopy (<1%), while 77% were found in the canopy and midstory, and only 22% of bees from this guild were sampled from the understory. Our findings are consistent with other studies that demonstrated a high abundance of wood‐nesting bees within the canopy (e.g., Campbell et al., 2018; Cunningham‐Minnick & Crist, 2020; Ulyshen et al., 2010; Urban‐Mead et al., 2021) and suggest that bees that nest in wood, including species that nest in moist decayed wood, or “soft” wood, exhibit a preference for canopy strata within forests likely due to the availability of wood‐nesting substrates such as dead limbs or knot holes. Available nesting substrate in the canopy has yet to be tested as a mechanism to explain the high abundance of wood‐nesting bees within the higher strata of forests since there is well‐documented availability of dead and rotting wood on the forest floor. Yet, there is a lack of correlation between coarse woody debris on the ground and the abundance of this guild in the canopy (Campbell et al., 2018; Ulyshen et al., 2010; Urban‐Mead et al., 2021). On the other hand, the hypothesis that native bees of temperate forests are abundant in the canopy strata during the summer due to the availability of alternative food sources has not been addressed either (Campbell et al., 2018; Ulyshen et al., 2010). Therefore, studies that quantify potential nesting substrates and alternative food sources for wood‐nesting bees within the canopy, including those that nest in “soft” wood, are clearly needed to resolve these discrepancies (Harmon‐Threatt, 2020).

Milam et al. (2022) found that the inclusion of canopy sampling in addition to understory sampling did not influence their ability to characterize the forest bee community. Our study supports their conclusion when only considering bees below the maximum height of the canopy (i.e., understory, midstory, and canopy strata) but further demonstrates that the bee community above the canopy is distinct from lower strata. The existence of bees above the forest canopy is highly relevant to understanding their ecology and may have additional implications for pollinator conservation vis a vis our understanding of the effects of habitat fragmentation and isolation on bee movements and related population processes throughout the landscape (Proesman et al., 2019; Roberts et al., 2017; Winfree et al., 2009). Though our study was limited in sampling intensity, it clearly demonstrates the complexities of spatiotemporal bee dynamics within forests, suggests a new perspective on the role of forests in the surrounding landscape, and emphasizes caution when drawing conclusions about forest bee communities that were sampled with vertically or temporally restricted designs. Thus, our study supports the growing body of literature that asserts the need for additional baseline research of forest bee communities along the full vertical gradient to inform forest management and bee conservation.

## AUTHOR CONTRIBUTIONS

**Michael J. Cunningham‐Minnick:** Conceptualization (lead); data curation (equal); formal analysis (lead); funding acquisition (lead); investigation (lead); methodology (lead); project administration (lead); resources (equal); software (lead); supervision (lead); visualization (lead); writing – original draft (lead); writing – review and editing (equal). **Joan Milam:** Conceptualization (supporting); data curation (equal); investigation (equal); methodology (supporting); resources (equal); supervision (supporting); writing – original draft (supporting); writing – review and editing (equal). **Brian Kane:** Conceptualization (supporting); data curation (supporting); investigation (equal); methodology (equal); resources (supporting); supervision (supporting); writing – original draft (supporting); writing – review and editing (equal). **H. Patrick Roberts:** Conceptualization (supporting); data curation (supporting); formal analysis (supporting); investigation (equal); methodology (supporting); writing – original draft (supporting); writing – review and editing (equal). **David I. King:** Conceptualization (supporting); investigation (equal); methodology (supporting); project administration (supporting); supervision (supporting); writing – original draft (supporting); writing – review and editing (equal).

## CONFLICT OF INTEREST

The authors declare no conflict of interest.

## Data Availability

The data that support the findings of this study are openly available in ScholarWorks, *Data and Datasets* at https://doi.org/10.7275/pmz5‐dn05.

## Appendix

**TABLE A1:** Location, diameter at breast height (DBH) in centimeters, and associated trap heights (m). Traps were hung 1 m above the tree canopy.

Site	Tree	Lat (°N)	Long (°W)	DBH	Understory	Midstory	Canopy	Above canopy
1	*Quercus rubra*	42.39884	−72.52118	89.2	1.3	11.8	20.9	28.9
1	*Acer rubrum*	42.39870	−72.52127	73.2	1.4	13.0	21.5	31.2
2	*Q. rubra*	42.39275	−72.52200	51.6	1.4	9.0	18.8	30.8
2	*A. rubrum*	42.39297	−72.52212	56.4	1.2	12.1	20.2	30.2

**TABLE A2:** Total bees of each species found at each stratum within the forest. The number of females are in parentheses. Numbers do not include five *Bombus*, four *Lasioglossum*, and three *Melissodes* specimens that could not be identified as species due to body damage

Species	Understory	Midstory	Canopy	Above canopy
*Agapostemon sericeus* (Förster, 1771)		1 (1)	1 (1)	
*Agapostemon texanus* (Cresson, 1872)	1 (1)			2 (2)
*Agapostemon virescens* (Fabricius, 1775)		1 (1)	2 (2)	2 (2)
*Andrena barbilabris* (Kirby, 1802)		1 (1)		
*Andrena bisalicis* (Viereck, 1908)			1 (1)	
*Andrena carlini* (Cockerell, 1901)	2 (2)		1 (1)	3 (3)
*Andrena cornelli* (Viereck, 1907)	8 (5)	1 (0)	1 (1)	
*Andrena frigida* (Smith, 1853)				1 (1)
*Andrena imitatrix* (Cresson, 1872)	2 (1)	3 (1)		28 (0)
*Andrena mandibularis* (Robertson, 1892)	2 (1)	3 (0)	2 (0)	2 (0)
*Andrena milwaukeensis* (Graenicher, 1903)		1 (0)	1 (0)	
*Andrena miserabilis* (Cresson, 1872)			2 (0)	
*Andrena nasonii* (Robertson, 1895)	3 (2)			
*Andrena pruni* (Robertson, 1891)	1 (0)	1 (0)		
*Andrena robertsonii* (Dalla Torre, 1896)		1 (0)		
*Andrena rugosa* (Robertson, 1891)	2 (0)	1 (0)	4 (4)	
*Andrena tridens* (Robertson, 1902)	1 (1)	1 (0)		
*Anthidium oblongatum* (Illiger, 1806)				1 (1)
*Anthophora terminalis* (Cresson, 1869)	1 (0)			
*Apis mellifera* (Linnaeus, 1758)	2 (2)		3 (3)	24 (24)
*Augochlora pura* (Say, 1837)	12 (11)	18 (17)	31 (29)	1 (1)
*Augochlorella aurata* (Smith, 1853)		1 (1)	1 (1)	
*Bombus bimaculatus* (Cresson, 1863)	1 (0)	10 (6)	4 (1)	6 (4)
*Bombus fervidus* (Fabricius, 1798)				2 (2)
*Bombus griseocollis* (DeGeer, 1773)				7 (7)
*Bombus impatiens* (Cresson, 1863)	12 (12)	17 (17)	50 (50)	54 (53)
*Bombus perplexus* (Cresson, 1863)	2 (1)	9 (6)	9 (6)	1 (1)
*Bombus sandersoni* (Franklin, 1913)			3 (2)	
*Bombus vagans* (Smith, 1854)			4 (3)	
*Ceratina calcarata* (Robertson, 1900)	8 (4)	11 (9)	30 (26)	
*Ceratina dupla* (Say, 1837)			2 (2)	
*Coelioxys moesta* (Smith, 1854)		1 (1)		
*Eucera pruinosa* (Say, 1837)				4 (4)
*Halictus ligatus* (Say, 1837)		1 (1)		
*Halictus parallelus* (Say, 1837)				1 (1)
*Halictus rubicundus* (Christ, 1791)		1 (1)	1 (0)	
*Hylaeus affinis/modestus*			1 (1)	
*Hylaeus sp. A/illinoiensis*		1 (0)		
*Lasioglossum bruneri* (Crawford, 1902)	1 (1)	2 (2)		
*Lasioglossum cinctipes* (Provancher, 1888)			1 (1)	1 (1)
*Lasioglossum coeruleum* (Robertson, 1893)	4 (4)	22 (22)	15 (15)	
*Lasioglossum coriaceum* (Smith, 1853)	6 (6)	8 (8)	5 (5)	
*Lasioglossum cressonii* (Robertson, 1890)		3 (2)	1 (1)	
*Lasioglossum foxii* (Robertson, 1895)	2 (2)			
*Lasioglossum hitchensi* (Gibbs, 2012)	1 (1)			
*Lasioglossum imitatum* (Smith, 1853)				1 (1)
*Lasioglossum lineatulum* (Crawford, 1906)				1 (1)
*Lasioglossum nigroviride* (Graenicher, 1911)	2 (2)	2 (2)		
*Lasioglossum pectorale* (Smith, 1853)			1 (1)	
*Lasioglossum pilosum* (Smith, 1853)				5 (5)
*Lasioglossum quebecense* (Crawford, 1907)	20 (20)	24 (22)	5 (4)	1 (1)
*Lasioglossum smilacinae* (Robertson, 1897)		1 (1)		
*Lasioglossum subviridatum* (Cockerell, 1938)	6 (6)		5 (5)	
*Lasioglossum versans* (Lovell, 1905)	1 (1)			
*Lasioglossum viridatum* (Lovell, 1905)	1 (1)			
*Lasioglossum weemsi* (Mitchell, 1960)	1 (1)			
*Megachile campanulae* (Robertson, 1903)			1 (0)	
*Megachile mendica* (Cresson, 1878)		1 (0)		
*Megachile montivaga* (Cresson, 1878)			1 (1)	
*Megachile rotundata* (Fabricius, 1793)				1 (0)
*Megachile sculpturalis* (Smith, 1853)				1 (1)
*Melissodes bimaculata* (Lepeltier de Saint Fargeau, 1825)				8 (8)
*Melissodes desponsus* (Smith, 1854)	3 (3)			1 (1)
*Melissodes trinodis* (Robertson, 1901)			1 (1)	
*Nomada armatella* (Cockerell, 1903)	1 (0)			
*Nomada* (bidentate‐group)	2 (2)		1 (1)	
*Nomada composita* (Mitchell, 1962)			1 (1)	
*Nomada luteoloides* (Robertson, 1895)	1 (1)		1 (0)	
*Osmia atriventris* (Cresson, 1864)	6 (3)	1 (1)		
*Osmia bucephala* (Cresson, 1864)	1 (1)			1 (1)
*Osmia cornifrons* (Radoszkowski, 1887)	9 (1)		1 (1)	
*Osmia pumila* (Cresson, 1864)	12 (10)	17 (14)	3 (3)	1 (0)
*Osmia sandhouseae* (Mitchell, 1927)	1 (0)			
*Osmia taurus* (Smith, 1873)	1 (1)			
*Xylocopa virginica* (Linnaeus, 1771)				1 (1)
TOTALS	142 (110)	166 (137)	197 (174)	162 (127)

**TABLE A3:** Test statistic (Pseudo‐*F*) on one degree of freedom from simulated contrasts of species composition between strata with associated *p*‐value adjusted for multiple comparisons.

Comparison	Pseudo‐*F*	*p*‐Value
Understory—Midstory	1.03	NS
Understory—Canopy	1.09	NS
Understory—Above Canopy	4.96	<.01
Midstory—Canopy	1.36	NS
Midstory—Above Canopy	5.87	<.01
Canopy—Above Canopy	3.34	<.01

## References

[ece39739-bib-0001] Allen, G. , & Davies, R. G. (2022). Canopy sampling reveals hidden potential value of woodland trees for wild bee assemblages. Insect Conservation and Diversity, 1–14. 10.1111/icad.12606

[ece39739-bib-0002] Arbizu, P. M. (2017). pairwiseAdonis: Pairwise multilevel comparison using Adonis . R package version 0.0.1.

[ece39739-bib-0003] Ascher, J. S. , & Pickering, J. (2020). Discover life bee species guide and world checklist (Hymenoptera: Apoidea: Anthophila) . http://www.discoverlife.org/mp/20q?/guide=Apoidea_species

[ece39739-bib-0004] Batra, S. W. T. (1985). Red maple (*Acer rubrum* L.), an important early spring food resource for honey bees and other insects. Journal of the Kansas Entomological Society, 58(1), 169–172.

[ece39739-bib-0005] Brooks, M. E. , Kristensen, K. , van Benthem, K. J. , Magnusson, A. , Berg, C. W. , Nielsen, A. , Skaug, H. J. , Maechler, M. , & Bolker, B. M. (2017). glmmTMB balances speed and flexibility among packages for zero‐inflated generalized linear mixed modeling. The R Journal, 9(2), 378–400.

[ece39739-bib-0006] Campbell, J. W. , Vigueira, P. A. , Viguiera, C. C. , & Greenberg, C. H. (2018). The effects of repeated prescribed fire and thinning on bees, wasps, and other flower visitors in the understory and midstory of a temperate forest in North Carolina. Forest Science, 64(3), 299–306.

[ece39739-bib-0007] Cannon, C. H. , Borchetta, C. , Anderson, D. L. , Arellano, G. , Barker, M. , Charron, G. , LaMontagne, J. M. , Richards, J. H. , Abercrombie, E. , Banin, L. F. , Casapia, X. T. , Chen, X. , Degtjarenko, P. , Dell, J. E. , Durden, D. , Andino, J. E. G. , Hernández‐Gutiérrez, R. , Hirons, A. D. , Kua, C. , … Spenko, M. (2021). Extending our scientific reach in arboreal ecosystems for research and management. Frontiers in Forests and Global Change, 4, 712165.

[ece39739-bib-0008] Cunningham‐Minnick, M. J. , & Crist, T. O. (2020). Floral resources of an invasive shrub alter native bee communities at different vertical strata in forest‐edge habitat. Biological Invasions, 22, 2283–2298.

[ece39739-bib-0009] Cunningham‐Minnick, M. J. , Roberts, H. P. , Kane, B. , Milam, J. , & King, D. I. (2022). A cost‐effective method to passively sample communities at the forest canopy‐aerosphere interface. Methods in Ecology and Evolution, 13, 2389–2396. 10.1002/ece3.9739 PMC9929519 36818539

[ece39739-bib-0010] Gibbs, J. (2011). Revision of the metallic Lasioglossum (Dialictus) of eastern North America (Hymenoptera: Halictidae: Halictini). Zootaxa, 3073, 1–216.

[ece39739-bib-0011] Gibbs, J. , Paker, L. , Dumesh, S. , & Danforth, B. N. (2013). Revision and reclassification of Lasioglossum (Evylaeus), L. (Hemihalictus) and L. (Sphecodogastra) in eastern North America (Hymenoptera: Apoidea: Halictidae). Zootaxa, 3672, 1–117. 26146702 10.11646/zootaxa.3672.1.1

[ece39739-bib-0012] Goulson, D. , Sangster, E. L. , & Young, J. C. (2011). Evidence for hilltopping in bumblebees? Ecological Entomology, 36, 560–563.

[ece39739-bib-0013] Harmon‐Threatt, A. (2020). Influence of nesting characteristics on health of wild bee communities. Annual Review of Entomology, 65, 39–56. 10.1146/annurev-ento-011019-024955 31923377

[ece39739-bib-0014] Harrison, T. , Gibbs, J. , & Winfree, R. (2018). Forest bees are replaced in agricultural and urban landscapes by native species with different phenologies and life‐history traits. Global Change Biology, 24, 287–296. 28976620 10.1111/gcb.13921

[ece39739-bib-0015] Hartig, F. (2020). DHARMa: Residual diagnostics for hierarchical (multi‐level / mixed) regression models . R package version 0.3.3.0. https://CRAN.R‐project.org/package=DHARMa

[ece39739-bib-0016] Hothorn, T. , Bretz, F. , & Westfall, P. (2008). Simultaneous inference in general parametric models. Biometrical Journal, 50(3), 346–363. 18481363 10.1002/bimj.200810425

[ece39739-bib-0017] Kebler, A. , & Somanathan, H. (2019). Spatial vision and visually guided behavior in Apidae. Insects, 10, 418. 31766747 10.3390/insects10120418 PMC6956220

[ece39739-bib-0018] LaBerge, W. E. (1987). A revision of the bees of the genus Andrena of the Western hemisphere. Part XII. Subgenera Leucandrena, Ptilandrena, Scoliandrena, and Melandrena. Transactions of the American Entomological Society, 112, 191–248.

[ece39739-bib-0019] LaBerge, W. E. (1989). A revision of the bees of the genus Andrena of the Western hemisphere. Part XIII. Subgenera Simandrena and Taeniandrena. Transactions of the American Entomological Society, 116, 1–56.

[ece39739-bib-0020] MacIvor, J. S. , Cabral, J. M. , & Packer, L. (2014). Pollen specialization by solitary bees in an urban landscape. Urban Ecosystem, 17(1), 139–147.

[ece39739-bib-0021] Maguire, D. Y. , Robert, K. , Brochu, K. , Larrivée, M. , Buddle, C. M. , & Wheeler, T. A. (2014). Vertical stratification of beetles (coleoptera) and flies (Diptera) in temperate forest canopies. Environmental Entomology, 43(1), 9–17. 24472199 10.1603/EN13056

[ece39739-bib-0022] Milam, J. , Cunningham‐Minnick, M. , Roberts, H. P. , Buelow, C. , & King, D. I. (2022). The contribution of canopy samples to assessments of forestry effects on native bees. Conservation Science and Practice, 4, e12690.

[ece39739-bib-0023] Mitchell, T. B. (1960). Bees of the eastern United States (Vol. 1). North Carolina Agricultural Experiment Station.

[ece39739-bib-0024] Mitchell, T. B. (1962). Bees of the eastern United States (Vol. 2). North Carolina Agricultural Experiment Station.

[ece39739-bib-0025] Nakamura, A. , Kitching, R. L. , Cao, M. , Creedy, T. J. , Fayle, T. M. , Freiberg, M. , Hewitt, C. N. , Itioka, T. , Koh, L. P. , Ma, K. , Malhi, Y. , Mitchell, A. , Novotny, V. , Ozanne, C. M. P. , Song, L. , Wang, H. , & Ashton, L. A. (2017). Forests and their canopies: Achievements and horizons in canopy science. Trends in Ecology & Evolution, 32(6), 438–451. 28359572 10.1016/j.tree.2017.02.020

[ece39739-bib-0026] Oksanen, J. , Blanchet, F. G. , Friendly, M. , Kindt, R. , Legendre, P. , McGlinn, D. , Minchin, P. R. , O'Hara, R. B. , Simpson, G. L. , Solymos, P. , Stevens, M. H. H. , Szoecs, E. , & Wagner, H. (2019). Vegan: Community ecology package . R package version 2.5‐6. https://CRAN.R‐project.org/package=vegan

[ece39739-bib-0027] Prendergast, K. S. , Menz, M. H. M. , Dixon, K. W. , & Bateman, P. W. (2020). The relative performance of sampling methods for native bees: An empirical test and review of the literature. Ecosphere, 11(5), e03076.

[ece39739-bib-0028] Proesman, W. , Bonte, D. , Smagghe, G. , Meeus, I. , & Verheyen, K. (2019). Importance of forest fragments as pollinator habitat varies with season and guild. Basic and Applied Ecology, 34, 95–107.

[ece39739-bib-0029] R Core Team . (2021). R: A language and environment for statistical computing. R Foundation for Statistical Computing. https://www.R‐project.org/

[ece39739-bib-0030] Roberts, H. P. , King, D. I. , & Milam, J. (2017). Factors affecting bee communities in forest openings and adjacent mature forest. Forest Ecology and Management, 394, 111–122.

[ece39739-bib-0031] Roubik, D. W. (1993). Tropical pollinators in the canopy and understory: Field data and theory for stratum “preferences”. Journal of Insect Behavior, 6(6), 659–673.

[ece39739-bib-0032] Russo, L. , & Danforth, B. (2017). Pollen preferences among the bee species visiting apple (*Malus pumila*) in New York. Apidologie, 48, 806–820.

[ece39739-bib-0033] Ruttner, F. (1966). The life and flight activity of drones. Bee World, 47, 93–100.

[ece39739-bib-0034] Smith, C. , Weinman, L. , Gibbs, J. , & Winfree, R. (2019). Specialist foragers in forest bee communities are small, social or emerge early. Journal of Animal Ecology, 88, 1158–1167. 31063228 10.1111/1365-2656.13003

[ece39739-bib-0035] Sobek, S. , Tscharntke, T. , Scherber, C. , Schiele, S. , & Steffan‐Dewenter, I. (2009). Canopy vs. understory: Does tree diversity affect bee and wasp communities and their natural enemies across forest strata? Forest Ecology and Management, 258, 609–615.

[ece39739-bib-0036] Ulyshen, M. D. , Soon, V. , & Hanula, J. L. (2010). On the vertical distribution of bees in a temperate deciduous forest. Insect Conservation and Diversity, 3, 222–228.

[ece39739-bib-0037] Urban‐Mead, K. R. , Muñiz, P. , Gillung, J. , Espinoza, A. , Fordyce, R. , van Dyke, M. , McArt, S. H. , & Danforth, B. N. (2021). Bees in the trees: Diverse spring fauna in temperate forest edge canopies. Forest Ecology and Management, 482, 118903.

[ece39739-bib-0038] Urban‐Mead, K. R. , Walter, E. , McArt, S. H. , & Danforth, B. N. (2022). Nearly half of spring‐flying male *Andrena* bees consume pollen, but less than female conspecifics. Apidologie, 53, 49.

[ece39739-bib-0039] Wainwright, C. E. , Stepanian, M. , Reynolds, D. R. , & Reynolds, A. M. (2017). The movement of small insects in the convective boundary layer: Linking patterns to processes. Scientific Reports, 7, 5438. 28710446 10.1038/s41598-017-04503-0 PMC5511248

[ece39739-bib-0040] Winfree, R. , Aguilar, R. , Vázquez, D. , LeHuhn, G. , & Aizen, M. (2009). A meta‐analysis of bees' responses to anthropogenic disturbance. Ecology, 90(8), 2068–2076. 19739369 10.1890/08-1245.1

[ece39739-bib-0041] Wood, T. J. , Gibbs, J. , Rothwell, N. , Wilson, J. K. , Gut, L. , Brokaw, J. , & Isaacs, R. (2018). Limited phenological and dietary overlap between bee communities in spring flowering crops and herbaceous enhancements. Ecological Applications, 28(7), 1924–1934. 30184292 10.1002/eap.1789

